# Supplementary material for: Modelling COVID-19 transmission in a hemodialysis centre using simulation generated contacts matrices
Source: PLoS One. 2021 Nov 19;16(11):e0259970. doi: 10.1371/journal.pone.0259970 (PMC8604317; doi:10.1371/journal.pone.0259970)
Supplement: Attachment — Submitted filename: Response to Reviewers.docx [file pone.0259970.s001.docx]

**Response to the Reviewers’ Comments**

on the Manuscript Entitled

“Modelling COVID -19 transmission in a hemodialysis centre using simulation generated

contacts matrices”
Submitted for publication in PLOS ONE [PONE-D-21-07385]

Mar 5, 2021

We are grateful to the Editor and to the Reviewers for carefully reviewing our paper, which has been revised according to your helpful comments. We believe that your suggestions have enhanced the quality of our paper. Our responses and revisions are given below, with the editor’s and reviewers’ original comments in italics.

**Review Comments to the Author**

**Reviewer #1:** *This very interesting paper provides relevant results regarding the operations of a typical large dialysis unit and generate contact matrices to examine outbreak scenarios
during COVID-19 pandemic. The authors presented the details of the contact matrix generation process and demonstrate how the simulation calculates a micro-scale contact matrix comprising the number and duration of contacts at a micro-scale time step. They used the contacts matrix in an agent-based model to predict disease transmission under different scenarios. The results show that micro-simulation can be used to estimate contact matrices, which can be used effectively for disease modelling in dialysis and similar settings. The manuscript is well written and organized. Methods are appropriate, results are clearly described and illustrated, as well as properly discussed. References are relevant and updated; however it could be useful to discuss the impact of cardiovascular risk factors in chronic kidney disease during COVID-19 pandemic, referring to the following articles doi:*[*10.31083/j.rcm.2019.04.548*](https://rcm.imrpress.com/EN/10.31083/j.rcm.2019.04.548)*,* [*https://doi.org/10.1159/000514467*](https://doi.org/10.1159/000514467)*. Therefore, this paper requires minor corrections and can be very useful for Plos One readers, because it provides very interesting information within the current context of published studies.*

**Response:**

Thank you for these affirmative and constructive comments. The mentioned impact has been highlighted and referenced in lines 82 - 84 of the revised version as follows**:**

**“**Furthermore, chronic kidney disease (CKD) can be considered as a cardiovascular risk equivalent, and COVID-19 that enhances the risk of cardiovascular events may synergize with pre-existing cardiovascular risk factors in CKD patients [6, 7].”

**Reviewer #2:** *I think the article is of interest and reflects the classic canons. demonstrates the goal set by the study. I will hence suggest the manuscript in this form. It respects research and publication ethics.*

**Response:**

Thank you for the affirmative and supportive comment.
